# Comprehensive characterization of 21-hydroxylase deficiency in a Chinese pediatric cohort: phenotype, steroid profiles and genetics

**DOI:** 10.3389/fendo.2025.1665306

**Published:** 2025-10-16

**Authors:** Hemeng Chong, Guocui Xue, Yalei Pi, Yanan Zhang, Yuqian Li, Yutong Xing, Baorong Chen, Huifeng Zhang

**Affiliations:** ^1^ Department of Pediatrics, The Second Hospital of Hebei Medical University, Shijiazhuang, China; ^2^ Kingmed Diagnostics (Beijing) Co., Ltd, Beijing, China

**Keywords:** 21-hydroxylase deficiency, steroid hormones, newborn screening, genotype, CAH-X syndrome

## Abstract

**Objective:**

This study aimed to investigate the clinical, steroid hormones and genetic characteristics of Chinese children with 21-hydroxylase deficiency (21OHD).

**Methods:**

This retrospective study included 115 children with 21OHD. Clinical data, steroid hormone levels, and genetic information were collected for overall and subgroup analyses. Clinical and steroid hormone characteristics were compared across clinical phenotypes and by sex. Within the salt-wasting (SW) group, characteristics were also compared between newborn screening (NBS)-diagnosed and clinically diagnosed patients. The relationship between Prader scores and both clinical phenotype and steroid hormone levels was analyzed, and the genotype-phenotype correlation, variation frequency and CAH-X CH-1 incidence were calculated.

**Results:**

The cohort comprised 76 (66.09%) SW, 27 (23.48%) simple virilizing (SV), and 12 (10.43%) non-classic (NC) patients. The overall levels of adrenocorticotropic hormone (ACTH), 17-hydroxyprogesterone(17OHP), and progesterone (P) levels were significantly elevated in SW and SV compared to NC patients. Girls with Prader scores ≥1 had higher hormone levels than those with normal external genitalia. Virilization was more severe in SW than SV girls. NBS significantly reduced the diagnostic delay for SW infants. Functional assays and SpliceAI prediction confirmed that a novel splice variant (c.203-1G>A) induces exon 2 skipping. We also report the first instance of cis double mutations (E3del8bp and p.V282L) on a single allele in two brothers. The allele frequency of p.V282L (5.16%) and CAH-X CH-1 incidence (11.32%) were higher than previously reported.

**Conclusion:**

This study expands the 21OHD mutational spectrum with two novel findings: a pathogenic splice-site variant (c.203-1G>A) and cis double mutations on a single allele. We demonstrate phenotype-specific differences in virilization and steroid hormones, underscoring the value of Prader scoring. NBS facilitated earlier diagnosis in SW patients, supporting its nationwide implementation in China. Our findings illustrate a distinct genetic architecture for 21OHD in the Chinese population, correlating with increased detection of NC cases.

## 1 Introduction

21-hydroxylase deficiency (21OHD)(OMIM: 201910) is an autosomal recessive disorder caused by pathogenic variants in the *CYP21A2* gene, accounting for over 90% of congenital adrenal hyperplasia (CAH) cases (1). The clinical phenotype correlates with the residual 21-hydroxylase activity: salt-wasting (SW) (minimal/no activity), simple virilizing (SV) (moderate deficiency), and non-classic (NC) (mild impairment) (2–4). 21OHD disrupts adrenal steroidogenesis, leading to distinct hormone profiles across phenotypes and sexes.

Elevated 17-hydroxyprogesterone (17OHP) is a sensitive diagnostic biomarker for 21OHD. Newborn screening (NBS) for 17OHP has been progressively implemented across most cities since 2018 in Hebei Province, China. NBS has been shown to reduce hospitalization and prevent morbidity and mortality from adrenal crises (5, 6). However, province-specific outcome data remain limited.

The *CYP21A2* gene resides at 6p21.3 within the RCCX module, a tandem arrangement of genes (*RP1-C4A-CYP21A1P-TNXA-RP2-C4B-CYP21A2-TNXB*) prone to recombination due to high homology between functional genes (*CYP21A2, TNXB*) and pseudogenes (*CYP21A1P*, *TNXA*) (5–7). These rearrangements generate pathogenic chimeric genes, complicating molecular diagnosis, which typically combines Sanger sequencing and multiplex ligation-dependent probe amplification (MLPA) (1, 8–10). Co-occurring *CYP21A2* and *TNXB* variants cause CAH-X syndrome, presenting with combined 21OHD and Ehlers-Danlos syndrome (EDS) features (~10% of 21OHD cases) (8, 11, 12). CAH-X syndrome is classified into three subtypes based on breakpoints within the chimeric *TNXA/TNXB* gene: CH-1: 120-bp deletion in exon 35; CH-2: Pathogenic variants disrupting exon 40; CH-3: Multiple clustered variants in exons 41 and 43 (11–14).

In this study, we comprehensively analyzed the clinical phenotype, steroid hormone profiles, and genetic spectrum in 115 Chinese children with 21OHD and functionally validated a novel pathogenic variant.

## 2 Materials and methods

### 2.1 Case data

#### 2.1.1 Study population and diagnostic criteria

We conducted a retrospective cohort study of children with suspected congenital adrenal hyperplasia (CAH) evaluated at the Second Hospital of Hebei Medical University from 2012 to 2023 (**Supplementary Figure S1**). A cohort of 119 cases with clinical and biochemical features of CAH—including hyperpigmentation, hypocortisolism, elevated ACTH, vomiting, atypical genitalia—along with elevated 17-OHP was collected. Four cases presenting hypertension or hypokalemia were excluded; all were confirmed by genetic testing to carry compound heterozygous mutations in *CYP11B1*.

Among the remaining cases, 106 underwent *CYP21A2* sequencing and MLPA analysis. Two carried only a single heterozygous mutation (one SW and one NC), while 104 had homozygous or compound heterozygous mutations, which were classified as SW (n = 71), simple virilizing (SV) (n = 25), or NC (n = 8) based on clinical and electrolyte profiles. The remaining nine cases, without genetic testing, were phenotypically categorized as SW (n = 4), SV (n = 2), or NC (n = 3) (Detailed clinical data are provided in **Supplementary Table S1**).

This retrospective analysis study was approved by the institutional ethics committee of the Second Hospital of Hebei Medical University (2023-R658). The study adhered to the Helsinki Declaration. Informed consent to participate was obtained from all of the participants’ guardians in the study.

#### 2.1.2 Phenotypic classification

115 patients were stratified into three clinical phenotypes based on established criteria:

- SW: Characterized by hyponatremia, hyperkalemia, dehydration, and atypical genitalia in girls.
- SV: Presenting with atypical genitalia at birth and progressive postnatal virilization in girls and peripheral precocious puberty in boys, both in the absence of salt-wasting symptoms.
- NC: Manifesting later-onset symptoms including premature pubarche, menstrual irregularities, or accelerated bone age.

#### 2.1.3 Newborn screening

NBS was performed using dried blood spots collected from heel prick 72 hours after birth, once adequate breastfeeding had been established. 17OHP was measured at designated screening centers using Time-Resolved Fluoroimmunoassay (TRFIA). Patients identified through NBS were classified as SW if they presented with hyponatremia and/or hyperkalemia and carried severe mutations (Group 0 or A). Those without electrolyte imbalance or elevated renin, and carrying partial-loss mutations, were classified as SV.

#### 2.1.4 Data collection

Comprehensive clinical and biochemical parameters were obtained at diagnosis:

- Demographic characteristics (sex, age at symptom onset and diagnosis).
- Anthropometric measurements (height, weight, bone age).
- Serum electrolytes (Na^+^, K^+^).
- Steroid hormone profiles (ACTH, cortisol, 17OHP, T, P, Androstenedione (AD), 21-Deoxycortisol (21-DOF), Dehydroepiandrosterone sulfate (DHEA-S)).
- Gonadotropin levels (follicle-stimulating hormone (FSH), luteinizing hormone (LH)).
- For female patients: Prader score and surgical history.
- Neonatal 17OHP screening results (if available).

### 2.2 Genetic testing

Peripheral blood was collected from the patients and their parents and genomic DNA was extracted, following the procedure of the DNA isolation system (Lab-Aid 820, Zeesan). First, MLPA was performed using the P050-D1CAH kit (MRC Netherlands, Amsterdam, The Netherlands) to detect large block deletions/transformations on a genetic analysis system. The MLPA contains six probes targeting the *TNXB* gene to detect the deletion of exon 35 of the *TNXB* gene. Then, PCR-specific amplification of the *CYP21A2* gene was performed with ME0008 and ME0066, followed by direct sequencing of the entire *CYP21A2* gene using a sequencer (3500xL Dx, Thermo Fisher, Inc.). The *CYP21A2* gene amplification failed in patients harboring large deletions/conversions on both alleles.

### 2.3 Genotype-phenotype correlation analysis

Patients were stratified into five genotype groups based on predicted residual 21-hydroxylase activity:

- Group 0: Complete enzyme inactivation caused by biallelic null variants (deletion, E3del8bp, E6 cluster, p.L308Ffs*6, p.Q319X, p.R357W) (14, 15).
- Group A: Minimal activity (homozygous I2G or compound heterozygous with null variants) (16).
- Group B: ~2–5% activity (homozygous p.I173N/p.R484P/p.R436C/p.R460H or compound heterozygous with Groups 0/A mutations) (17).
- Group C: 20–50% activity (homozygous p.V282L/p.P31L or compound heterozygous with Groups 0/A/B variants) (18–20).
- Group D:Patients with novel or biochemically-not-assessed mutations (21).

Patients in Groups 0 and A were predicted to have a phenotype of SW, patients in Group B were predicted SV phenotype, and NC phenotype for Group C patients. As the observed clinical phenotype did not always match the genotype-predicted phenotype within each group, the positive predictive value (PPV) was calculated to quantify this correlation. PPV was defined as: (Number of patients with the expected phenotype in the group/Total patients in the group) × 100%.

### 2.4 Analysis of the pathogenicity of the novel c.203-1G>A mutation in *CYP21A2*

#### 2.4.1 *In silico* pathogenicity prediction

Splice-altering potential was assessed using Splice-AI (https://spliceailookup.broadinstitute.org/).

#### 2.4.2 *In vitro* experiments

**Cell culture:** HEK293T and HeLa cells (Chinese Academy of Sciences Cell Bank) were maintained at 37 °C/5% CO_2_. The PCR primers used in follow procedure are shown in **Supplementary Table S1**.

**Plasmid construction**: We modified the pEGFP-N1 vector (Addgene #172281) by inserting a 3×FLAG tag sequence (after CMV promoter) and T2A (pre-EGFP) sequences. We then amplified the wild-type *CYP21A2* fragment from HEK293T genomic DNA (TIANamp Kit) via nested PCR with flanking XhoI/KpnI sites. Subsequently, we generated the c.203-1G>A mutant using overlap extension PCR. Finally, we ligated the respective fragments (*CYP21A2*-WT or *CYP21A2*-MUT) into the modified vector backbone to create the constructs pCMV-CYP21A2-WT and pCMV-CYP21A2-MUT. Both constructs were verified by Sanger sequencing (for the full insert in WT and the specific mutation in MUT).

**Transfection and splicing analysis:** Plasmids (pCMV-CYP21A2-WT and pCMV-CYP21A2-MUT) were transfected into HEK293T and HeLa cells using Lipofectamine 2000. Total RNA was extracted from transfected cells 48–72 hours post-transfection (Omega Bio-Tek E.Z.N.A.^®^ Total RNA Kit I). cDNA was then synthesized from the extracted RNA (MonScript™ RTIII Super Mix with dsDNase). Transcripts encompassing the region of interest were amplified via nested PCR. The resulting PCR products were analyzed through 1% agarose gel electrophoresis. Bands corresponding to expected sizes were gel-purified (BioFlux Gel Extraction Kit), and finally subjected to bidirectional Sanger sequencing to determine the splicing pattern.

### 2.5 Statistical analysis

All statistical analyses were conducted using IBM SPSS Statistics (version 22.0). Due to non-normally distributed data, continuous variables are presented as median and interquartile range (IQR). Missing data were handled using multiple imputation: variables with missingness <15% (Na^+^, K^+^, ACTH, 17OHP, T, P) were imputed, while those with higher missing rates (AD, 18.26%; cortisol, 20.00%; 21DOF, 27.82%; DHEA-S,30.43%) were included only as predictors. Twenty imputed datasets were generated and pooled according to Rubin’s rules.

Group comparisons (e.g., SW vs SV, SV vs NC, SW vs NC; overall and sex-specific subgroups; NBS vs clinical diagnosis in SW; vulvar masculinization degree in girls) were performed using Mann–Whitney U test or chi-squared test, as appropriate. Bonferroni correction was applied for multiple comparisons. Statistical significance was defined as P < 0.05 (two-sided).

## 3 Results

### 3.1 Clinical data analysis

#### 3.1.1 Clinical characteristics by phenotype

The clinical characteristics and steroid hormone levels of 115 patients with 21OHD (SW = 76, SV = 27, NC = 12) at the time of initial diagnosis are shown in **Table 1**. Overall, compared to the combined SV and NC groups, patients with the SW form exhibited a younger age at symptom onset and/or diagnosis, a shorter interval from symptom onset to diagnosis, as well as significantly lower Na^+^ along with higher levels of K^+^, ACTH, 17OHP, T, and P. Furthermore, when comparing SV and NC patients, those with the SV form showed both an earlier age of onset and diagnosis in addition to elevated K^+^, ACTH, 17OHP, and P levels. P levels were significantly higher in SW and SV females—as well as in SW males—compared to their NC counterparts. No significant sex-based differences in steroid levels were detected within the same phenotypic subgroups. Females with classic 21OHD due to visible atypical genitalia present earlier than males, who often present later with salt-wasting crisis or precocious puberty. Conversely, among NC patients, males presented with an earlier onset and diagnosis than females.

**Table 1:** Phenotype- and sex-stratified profiles of the 115 Chinese children with 21OHD at initial diagnosis.

	SW			SV			NC		
	Male (n=47)	Female (n=29)	Total (n=76)	Male (n=11)	Female (n=16)	Total (n=27)	Male (n=6)	Female (n=6)	Total (n=12)
Sym age (yr)	0.03 (0.01;0.06) ^ac*^	0.00 (0.00;0.00) ^ac^	0.01 (0.00;0.04) ^ac^	4.42 (1.30;6.48) ^*^	0.00 (0.00;0.59) ^b^	0.18 (0.00;4.25) ^b^	3.96 (2.92;6.10) ^*^	11.85 (7.65;14.64)	7.43 (3.72;12.68)
Sym-diag interval (yr)	0.05 (0.02;0.09) ^ac^	0.06 (0.04;0.24) ^ac^	0.05 (0.02;0.11) ^ac^	0.60 (0.03;3.81)	2.04 (0.70;4.31)	1.83 (0.31;4.26)	3.81 (1.72;4.69)	1.23 (0.37;3.29)	2.72 (0.50;4.22)
Diag age (yr)	0.08 (0.05;0.14) ^ac^	0.06 (0.04;0.24) ^ac^	0.07 (0.05;0.16) ^ac^	6.51 (3.07;8.37)	2.78 (0.74;5.70) ^b^	4.32 (1.68;8.34) ^b^	7.24 (6.73;9.59) ^*^	13.49 (8.16;16.92)	8.91 (6.98;13.56)
Na+ (mmol/L)	119.40 (107.60; 126.50) ^ac^	122.60 (112.30; 131.50) ^ac^	120.75 (110.20; 130.03) ^ac^	138.00 (132.90; 138.80)	134.90 (123.71; 138.95)	137.00 (124.51; 138.80) ^b^	138.00 (102.45; 140.03)	140.15 (138.58; 142.80)	139.20 (137.25; 141.80)
K+ (mmol/L)	6.82 (5.93;7.89) ^ac^	6.30 (5.73;7.70) ^ac^	6.79 (5.80:7.85) ^ac^	4.56 (3.98;5.10)	4.79 (4.47;6.02) ^b^	4.78 (4.30;5.88) ^b^	4.15 (3.06;4.30)	4.12 (3.96;4.26)	4.15 (4.00;4.28)
ACTH (pg/ml)	230.00 (91.80; 524.00)	362.80 (128.15; 1072.55) ^a^	255.45 (107.09; 846.20) ^a^	102.00 (79.51; 374.60)	245.50 (81.35; 322.50)	141.00 (79.51; 328.00) ^b^	71.39 (22.75; 133.63)	79.70 (20.30; 124.50)	79.70 (22.25; 119.75)
17OHP (ng/ml)	127.70 (25.10; 232.10)	139.60 (76.90; 277.10)	130.05 (49.50; 251.08) ^a^	82.70 (71.95; 116.50)	80.25 (30.86; 186.42)	82.70 (34.20; 148.70) ^b^	19.50 (11.51; 66.43)	70.73 (2.99; 146.87)	37.00 (5.91: 89.84)
T (pg/ml)	3937.50 (1645.50; 5670.00)	4330.00 (2565.00; 8720.00)	4120.00 (1953.25; 7781.00) ^ac^	1730.00 (785.30; 3596.00)	2687.00 (1375.65; 4954.58)	2280.00 (1129.70; 3795.86)	926.09 (227.50; 3180.00)	1838.60 (265.28; 5392.50)	1499.04 (249.53; 3366.80)
P (ng/ml)	13.26 (6.95;18.74) ^a^	13.86 (7.38;24.76) ^a^	13.36 (7.37;21.03) ^a^	6.74 (1.40;14.96)	10.54 (7.15;15.90) ^b^	9.70 (5.04:14.96) ^b^	1.64 (0.27;5.31)	2.13 (0.91;6.87)	2.13 (0.37:4.28)

Different superscript letters (a, b, c) indicate statistically significant differences (*P* < 0.05) between phenotypes: a, SW vs NC; b, SV vs NC; c, SW vs SV. Asterisk (*) indicates significant difference (*P* < 0.05) between males and females within the same phenotype group.

Sym age, age at symptom onset; Sym-diag interval, symptom-to-diagnosis interval; Diag age, age at diagnosis; yr, years-old.

A sensitivity analysis comparing the results derived from multiply imputed data with those from complete case analysis (**Supplementary Table S2**) demonstrated closely matched medians and consistent statistical significance across the SW, SV, and NC groups, both in the overall cohort and in gender-specific subgroups. These findings suggest that the primary results are robust to the method of handling missing data.

#### 3.1.2 Comparison of NBS and clinically diagnosed patients

Among 115 patients, 33 (SW = 31, SV = 2) patients accepted NBS were reported abnormal 17OHP. Comparative analysis of SW patients diagnosed via abnormal NBS (n=31) versus clinical diagnosis (n=45) revealed key differences (**Table 2**). NBS significantly reduced the diagnostic delay by 50% (median: 18d vs. 27d; P = 0.02). Accordingly, the age at diagnosis was substantially earlier in the NBS group (median: 21d vs. 43d; P = 0.03). However, no significant differences were observed between the two groups in sex distribution, electrolyte levels, or steroid hormone concentrations at the time of diagnosis.

**Table 2:** Comparison of SW patient profiles by diagnostic pathway (NBS vs clinical presentation).

	Abnormal NBS (n=31)	Clinical diagnosis (n=45)	Z/χ²	P
Sym age (d)	7.00(0.00;20.00)	5.00(0.00;16.00)	0.28	0.78
Sym-diag interval (d)	18.00(7.00;27.00)	27.00(11.00;67.00)	2.41	0.02
Diag age (d)	23.00(19.00;38.00)	41.00(18.50;84.50)	2.19	0.03
Sex (n, %)				
Male	22/47(46.81%)	25/47(53.19%)	1.85	0.17
Female	9/29(31.03%)	20/29(68.97%)		
Na^+^ (mmol/L)	122.60(110.80;131.10)	119.90(109.50;129.20)	0.72	0.47
K^+^ (mmol/L)	6.58(5.77;8.35)	6.79(5.85;7.83)	0.37	0.72
ACTH (pg/ml)	132.00(79.38;892.30)	289.40(117.65;820.40)	1.46	0.14
17OHP (ng/ml)	127.70(63.07;264.00)	135.00(47.85;223.67)	0.01	1.00
T (pg/ml)	3277.00(1645.50;4778.80)	4323.46(2251.50;9025.00)	1.76	0.08
P (ng/ml)	13.19(6.36;14.24)	14.01(7.86;25.03)	1.73	0.08

Sym age, age at symptom onset; Sym-diag interval, symptom-to-diagnosis interval; Diag age, age at diagnosis; d, days old; Z/χ², the Z value (for Mann-Whitney U test) or the chi-square value (for the chi-square test).

#### 3.1.3 Virilization patterns in females with 21OHD

**Figure 1:**
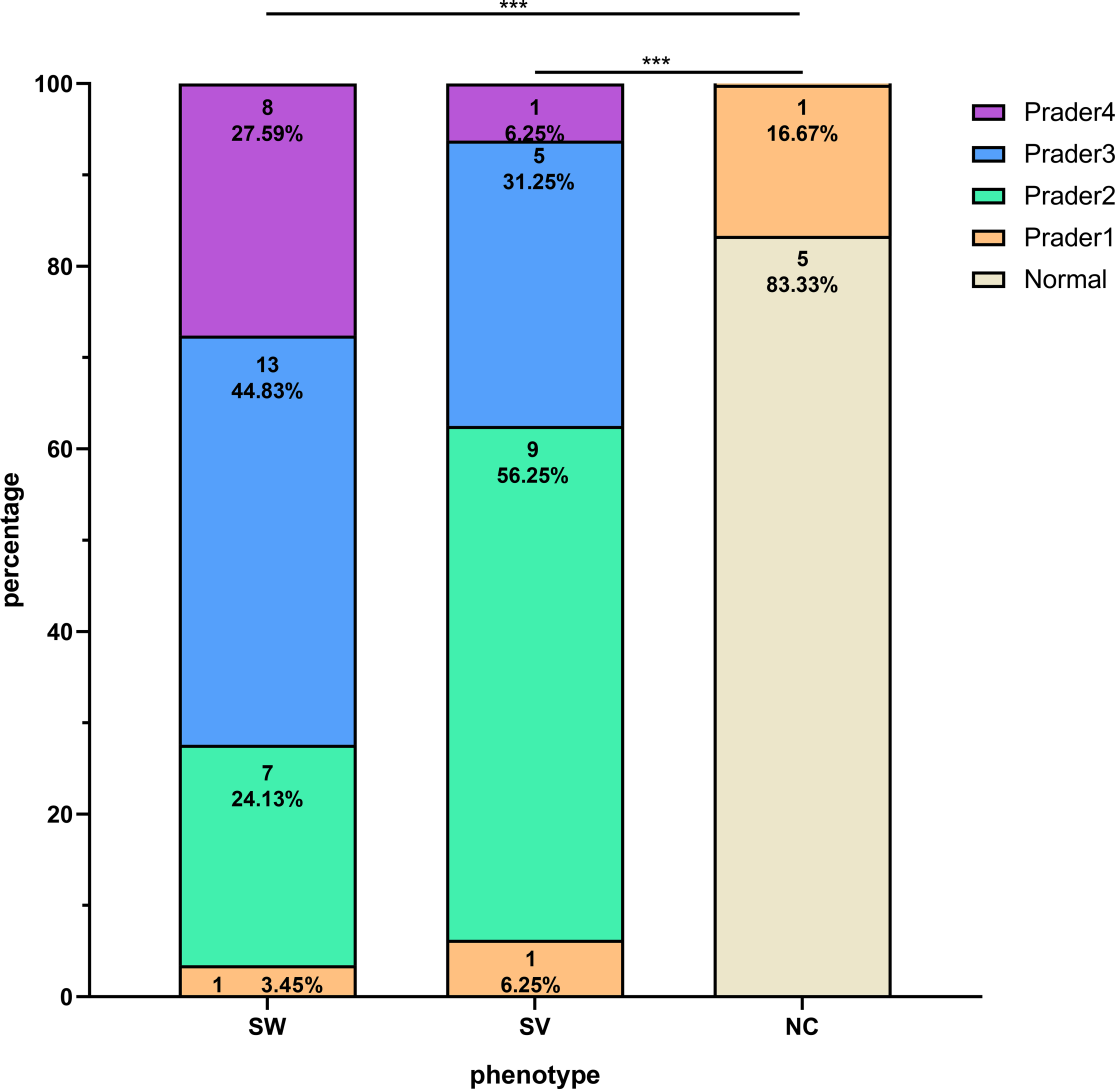
Distribution of Prader scores among girls with 21OHD stratified by clinical phenotypes. The symbol *** denotes statistical significance at p < 0.001.

As illustrated in **Figure 1**, the distribution of Prader scores varied markedly across the groups. Among the SW group (n=29), the majority were classified as Prader 3 (44.83%), whereas the SV group (n=16) was predominantly Prader 2 (56.25%). In contrast, the NC group (n=6) showed predominantly normal external genitalia (83.33%). Overall comparisons revealed statistically significant differences in composition ratios between the SW/SV and NC groups (P < 0.001). Furthermore, the proportion of individuals with Prader ≥3 was significantly higher in the SW group (72.42%) compared to the SV group (37.50%) (P = 0.022).

As summarized in **Table 3**, clinical and hormonal correlates also differed significantly according to Prader classification. Specifically, subjects with Prader ≥1 (n = 46) exhibited significantly earlier symptom onset and time of diagnosis (P < 0.001), along with higher ACTH, 17OHP, and progesterone levels (P < 0.05), compared to those with normal external genitalia (n = 5). Additionally, the surgery rate was markedly higher in the Prader ≥1 group (78.26% vs. 0.00%, P = 0.002). When comparing the Prader ≥3 (n = 27) and Prader 1~2 (n = 19) subgroups, the former was associated with a younger age at diagnosis and surgery (P < 0.01), a shorter diagnostic delay (P = 0.02), and a higher surgical rate (92.59% vs. 57.89%, P = 0.01).

**Table 3:** Clinical and biochemical profiles of 21OHD girls by Prader score.

	Normal external genitalia (n=5)	Prader≥1(n=46)					Z/χ²	P
		Prader 1~2(n=19)	Prader≥3(n=27)	Z/χ²	P	Total		
Sym age (y)	10.19(7.43;14.97)	0.00(0.00;0.78)	0.00(0.00;0.00)	2.61	0.01	0.00(0.00;0.00)	4.51	<0.001
Sym-diag Interval (y)	1.80(0.55;3.43)	1.54(0.11;4.26)	0.10(0.04;0.42)	2.34	0.02	0.19(0.05;1.84)	1.84	0.07
Diag age (y)	13.35(7.98;17.72)	2.66(0.14;5.80)	0.10(0.04;0.42)	2.90	0.004	0.20(0.05;2.72)	3.33	0.001
Surgery (n, %)	0/5(0.00%)	11/19(57.89%)	25/27(92.59%)	5.98	0.01	36/46(78.26%)	9.80	0.002
Surgery age (y)	–	3.80(2.20;5.30)	2.10(1.75;3.10)	2.62	0.01	2.45(1.80;3.55)	–	–
ACTH (pg/ml)	78.90(19.40;125.00)	227.00(75.70;459.00)	362.13(135.00;646.00)	1.41	0.16	267.40(109.26;595.08)	2.54	0.01
17OHP (ng/ml)	53.60(2.88;99.84)	122.80(33.13;226.13)	139.21(49.07;278.00)	0.50	0.62	137.87(39.08;255.00)	2.06	0.04
T (pg/ml)	1460(203.85;6268.60)	3750.00(1440.00;7230.00)	3710.00(2260.00;8540.00)	0.56	0.58	3730.00(2245.75;7377.50)	1.76	0.08
P (ng/ml)	2.02(0.63;3.31)	13.30(6.63;16.31)	13.83(8.21;25.02)	1.08	0.28	13.51(7.39;21.24)	3.58	<0.001

Z/χ², the Z value (for Mann-Whitney U test) or the chi-square value (for the chi-square test).

### 3.2 Genetic analysis

Molecular testing of *CYP21A2* was performed in 106 proband-parent trios. Biallelic pathogenic variants were identified in 104 patients (SW = 71, SV = 25, NC = 8), while two patients harbored single heterozygous mutations (I2G [NC phenotype] and p.Q319X [SW phenotype]). The remaining 9 patients did not undergo genetic testing and were diagnosed clinically based on biochemical and phenotypic evidence (SW = 4, SV = 2, NC = 3). The final cohort comprised 115 patients from 107 unrelated families (SW = 76, SV = 27, NC = 12).

Genotype-phenotype correlations were summarized in **Table 4**. PPV for each variant group was calculated as follows: Group 0 (null variants) exhibited a 100% PPV for the SW form, while Group A showed an 87.5% PPV for SW. Conversely, Group B demonstrated a 76.9% PPV for the SV form, and Group C had a 75.0% PPV for the NC form.

**Table 4:** Genotype-phenotype correlation in 106 patients with 21OHD who underwent genetic testing.

Genotype	Allele 1	Allele 2	Male			Female			Total	PPV
			SW	SV	NC	SW	SV	NC		
Group 0	Large del or con	Large del or con	2							
	Large del or con	p.Q319X				1				
	Large del or con	p.R357W	1			1				
	Large del or con	p.L308Ffs*6	2			1				
	Large del or con	E3del8bp	1			1				
	Large del or con	p.R484fs*40	2							
	p.R357W	E3del8bp	1							
	E3del8bp	p.L308Ffs*6	1							
	p.Q319X	p.Q319X				1				
									15	100%
Group A	I2G	Large del or con	10			4				
	I2G	I2G	5	1		1	1			
	I2G	p.Q319X	2			3				
	I2G	p.L308Ffs*6				1	2			
	I2G	p.R357W	3			2				
	I2G	E3del8bp	1							
	I2G	p.R484Pfs*58	1			1				
	I2G	E3del8bp & p.V282L	1	1						
									40	87.50%
Group B	p.I173N	Large del or con	1			1	5			
	p.I173N	p.I173N	1				1			
	p.I173N	I2G	1	4		1	6			
	p.I173N	E6cluster		1						
	p.I173N	p.Q319X				1	1			
	p.I173N	p.R357W		2						
									26	76.92%
Group C	p.P31L	Large del or con						1		
	p.V282L	Large del or con						1		
	p.V282L	p.I173N			1					
	p.V282L	I2G	2		1					
	p.V282L	p.V282L			1			1		
									8	75.00%
Group D	I2G	p.L322P				1				
	I2G	p.S216X				2				
	I2G	No pathologic mutations detected			1					
	p.Q319X	No pathologic mutations detected	1							
	p.Q319X	c.-113G>A	1							
	p.Q319X	p.R150P	1							
	p.Q319X	p.R484W						1		
	Large del or con	c.203-1G>A				1				
	Large del or con	p.G179R			1					
	Large del or con	p.A34D	1							
	Large del or con	p.R357Q	1							
	p.L308Ffs*6&p.Q319X	p.R342W	1							
	p.L308Ffs*6	p.G292S	2							
	E6cluster	p.R355C	2							
									17	

Del, deletion, con, conversion, &, *Cis* double mutations.

Discordant cases were, however, observed in each group. Group A included five SV patients with the following genotypes: I2G homozygotes (n=2), I2G/p.L308Ffs*6 (n=2), and I2G/E3del8bp & p.V282L (n=1). In contrast, six SW cases were found in Group B, including p.I173N homozygotes (n=1), p.I173N/Large del or con (n=2), p.I173N/I2G (n=2), and p.I173N/p.Q319X (n=1). Additionally, two SW patients were identified in Group C, both genotyped as p.V282L/I2G.

Notable genotypes included cis double mutations and *de novo* events. Cis double mutations occurred in three males: a paternal E3del8bp & p.V282L allele in two Group A brothers (**Supplementary Figure S2**), and a paternal p.L308Ffs*6 & p.Q319X allele in a Group D patient (**Supplementary Figure S3**). Additionally, *de novo* mutations were confirmed in three patients: p.I173N (female), p.R357W (male), and an exon 6 deletion (male) (**Supplementary Figure S4**).

Of the 30 patients harboring CYP21A2 deletions, 40% (12/30) exhibited co-occurring TNXB exon 35 deletions, confirming a diagnosis of CAH-X CH-1 (**Table 5**; **Supplementary Figure S5**).

**Table 5:** Co-occurrence of *TNXB* exon 35 deletions and *CYP21A2* deletions (CAH-X CH-1).

Patients(sex)	*CYP21A2* (allele1/allele2)	*TNXB* gene exon 35 deletion	Patients(sex)	*CYP21A2* (allele1/allele2)	*TNXB* gene exon 35 deletion
CH-1-01(F)	del/p.I173N	Yes	CH-1-16(M)	del/del	Yes
CH-1-02(F)	del/p.V282L	No	CH-1-17(M)	del/I2G	Yes
CH-1-03(F)	del/I2G	No	CH-1-18(F)	del/I2G	Yes
CH-1-04(M)	del/p.L308Ffs*6	No	CH-1-19(M)	del/I2G	No
CH-1-05(F)	del/p.I173N	No	CH-1-20(M)	del/I2G	No
CH-1-06(F)	del/p.P31L	No	CH-1-21(M)	del/p.R357W	No
CH-1-07(F)	del/p.I173N	No	CH-1-22(F)	del/p.L308Ffs*6	Yes
CH-1-08(F)	del/p.I173N	No	CH-1-23(M)	del/I2G	Yes
CH-1-09(F)	del/c.203-1G>A	Yes	CH-1-24(M)	del/I2G	Yes
CH-1-10(F)	del/p.I2G	No	CH-1-25(M)	del/(exon1-3del)	No
CH-1-11(M)	del/p.R484fs*40	No	CH-1-26(F)	del/I2G	Yes
CH-1-12(M)	del/p.G179R	Yes	CH-1-27(M)	del/p.L308Ffs*6	No
CH-1-13(M)	del/p.I173N	No	CH-1-28(M)	del/I2G	No
CH-1-14(M)	del/I2G	No	CH-1-29(F)	del/E3del8bp	Yes
CH-1-15(F)	del/p.I173N	No	CH-1-30(M)	del/E3del8bp	Yes

M, male, F, female.

#### 3.2.1 Variant spectrum of *CYP21A2*

The variant spectrum of CYP21A2 was characterized in detail. As summarized in **Table 6**, micro-conversions constituted the majority of allelic variants (70.89%, n=151), followed by large deletions or conversions (19.25%, n=41) and bona fide point mutations (9.86%, n=21). The most prevalent variant was I2G (31.46%), with large deletions/conversions (19.25%), p.I173N (13.62%), p.Q319X (7.04%), and p.V282L (5.16%) also being predominant.

**Table 6:** Spectrum and frequency of *CYP21A2* variants identified in 213 alleles from 106 patients with 21OHD.

Mutation type	Mutation on the DNA level	Mutation on protein level		Location on *CYP21A2* gene	Mutation type	Alleles	Relative frequency
Micro-conversions	c.293–13 A/C>G (I2G)			Intron 2	splicing	67	31.46%
	c.518 T > A		p.I173N	Exon 4	missense	29	13.62%
	c.1069 C>T		p.R357W	Exon 8	missense	10	4.69%
	c.955 C > T		p.Q319X	Exon 8	nonsense	15	7.04%
	c.844 G>T		p.V282L	Exon 7	missense	11	5.16%
	c.923dupT		p.L308Ffs*6	Exon 7	duplication	10	4.69%
	c.710 T > A; c.713 T > A; c.719 T > A		E6 cluster (p.I236N; p.V237E; p. M239K)	Exon 6	missense	3	1.41%
	c.92 C>T		p.P31L	Exon 1	missense	1	0.47%
	c.332_339del(E3del8bp)		p.G111Vfs*21	Exon 3	deletion	7	3.29%
Large deletion or crossover	deletion/conversion E1-7			Exon 1-7		31	14.55%
	deletion/conversion E1-3			Exon 1-3		8	3.76%
	deletion/conversion E1-6			Exon 1-6		1	0.47%
	deletion/conversion E6			Exon 6		1	0.47%
bona fide point mutations	c.1451_1452delGGinsC		p.R484Pfs*58	Exon 10	indel	2	0.94%
	c.965T>C		p.L322P	Exon 8	missense	1	0.47%
	c.874G>A		p.G292S	Exon 7	missense	2	0.94%
	c.1450C>T		p.R484W	Exon 10	missense	1	0.47%
	c.-113G>A			Promoter		1	0.47%
	c.449G>C		p.R150P	Exon 4	missense	1	0.47%
	c.203-1G>A		–	Intron 1	splicing	1	0.47%
	c.1450_1451 insC		p.R484fs*40	Exon 10	indel	2	0.94%
	c.535G>A		p.G179R	Exon 4	missense	1	0.47%
	c.377C>G		p.S126X	Exon 3	nonsense	2	0.94%
	c.1024C>T		p.R342W	Exon 8	missense	1	0.47%
	c.101C>A		p.A34D	Exon 1	missense	1	0.47%
	c.1070G>A		p.R357Q	Exon 8	missense	1	0.47%
	c.1063C>T		p.R355C	Exon 8	missense	2	0.94%
						213	

**Figure 2:**
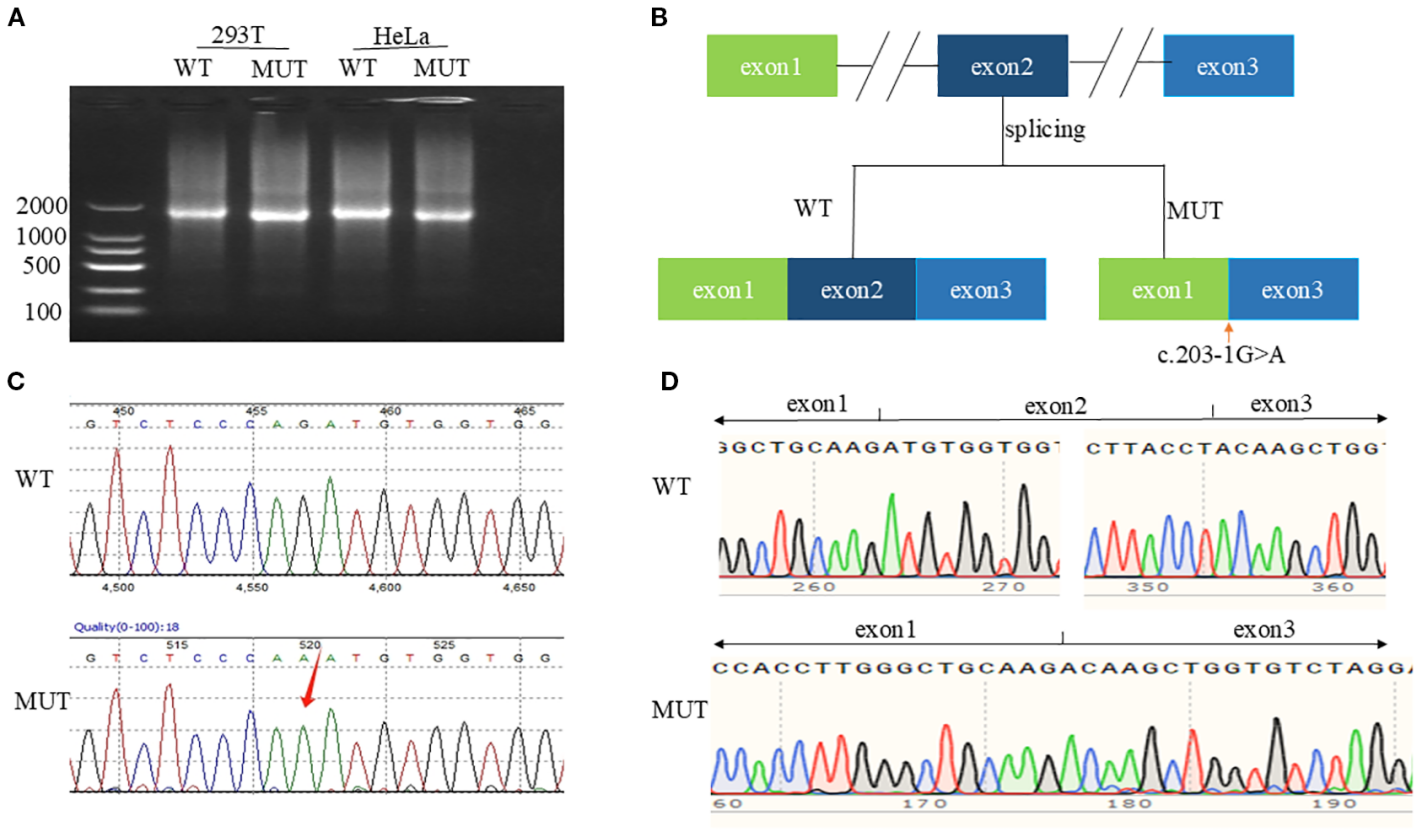
*In vitro* functional characterization of c.203-1G>A. **(A)** Agarose gel of RT-PCR products from cells transfected with WT or MUT CYP21A2 plasmids. **(B)** Splicing mechanism: WT (exon 1-2-3) vs. MUT (exon 2 skipping). **(C)** Genomic Sanger chromatogram showing heterozygous c.203-1G>A (arrow). **(D)** cDNA sequencing confirming exon 2 deletion (c.203_292del).

Bona fide point mutations consisted of nine missense, one nonsense, one splice-site, two indel, and one promoter variant. Recurrent mutational hotspots were observed, notably a missense cluster in exon 8 and indels targeting codon R484 in exon 10. Additionally, we identified a novel splice-site variant in intron 1, c.203-1G>A (**Figure 2C**, **Supplementary Figure S6a**).

#### 3.2.2 Analysis of pathogenicity of novel mutation c.203-1G>A

Splice-AI online tool predicted Splice Loss=0.99(1bp), and Splice Gain=0.15(-61bp), hypothesizing that the splice variant leads to a high probability of splice deletion as the wild-type motif “cccagATG” was changed to “cccaaATG” without uncovering a cryptic splice site.

*In vitro* functional validation experiments: Agarose gel electrophoresis showed that the target cDNAs detected in HEK293T and HeLa cells transfected with pCMV-CYP21A2-WT plasmid were both of higher molecular weight than those transfected with pCMV-CYP21A2-MUT plasmid (**Figure 2A**, **Supplementary Figure S7**). Sanger sequencing confirmed the exon 2 skipping in the mature mRNA (**Figures 2B, D**, **Supplementary Figure S6b**), and the protein was predicted to be truncated. The mutated mRNA is denoted as NM_000500.9: c.203_292del.

## 4 Discussion

This study advances beyond prior genetic-focused investigations of 21OHD (10, 21, 22) by integrating clinical, hormonal, and molecular profiling in 115 Chinese pediatric patients. We systematically compared phenotype-stratified clinical and steroid hormone characteristics, analyzed sex-specific differences, evaluated Prader score correlations with phenotypes/hormones in females, and quantified genotype-phenotype concordance, variant frequencies, and CAH-X CH-1 incidence.

Key clinical distinctions emerged at diagnosis: SW infants presented neonatally with classic electrolyte crises, enabling prompt diagnosis. SV children manifested early virilization but faced diagnostic delays (median diagnosis age: 4.32 years), reflecting low disease awareness for isolated masculinization. NC boys were typically identified during school-age growth assessments via advanced bone age—a consequence of increased developmental surveillance. Their historical underrepresentation in cohorts likely stems from underdiagnosis.NC girls presented peripubertally with menstrual irregularities ± virilization, aligning with existing reports (22). Simultaneously, the diagnosis of NC form also highlights the importance of genetic testing (23).

Steroid hormone analysis revealed significantly elevated levels of 17OHP and P in SW and SV patients compared to NC patients, consistent with enhanced ACTH stimulation. Sex-specific variations were observed: P levels were significantly higher in SW and SV females—as well as in SW males—compared to their NC counterparts. However, no significant sex-based differences in steroid levels were detected within the same phenotypic subgroups. In contrast, Chang et al. (24) reported sex-related hormonal differences within phenotypes—a discrepancy potentially attributable to our smaller sex-stratified subgroups or limited biomarker panel.

Notably, a male predominance (55.65%; 64/115) was observed, which was particularly pronounced in the SW subgroup (61.84%). This finding aligns with a Chinese cohort analysis by Xia et al. (22) and may be attributed to the fact that females with virilization often receive earlier diagnoses in primary care settings, while males with electrolyte crises require specialized referral to tertiary centers. Previous studies have reported that clinically diagnosed cohorts typically exhibit a female predominance (25, 26). Furthermore, a higher proportion of patients with severe genotypes were diagnosed after the implementation of NBS compared to the pre-screening era (27), highlighting significant pre-NBS mortality among severe phenotypes—especially males.

Critically, NBS significantly advanced the age at diagnosis for SW infants, although no significant differences were observed in electrolyte and biochemical parameters at the time of diagnosis, early detection mitigated long-term homeostatic imbalances and associated physiological damage, particularly for SW males. Family histories revealed neonatal deaths in undiagnosed cases, emphasizing NBS’s role in preventing mortality.

Female patients exhibited distinct virilization patterns across phenotypes: Prader 3 (44.83%) predominated in SW, while Prader 2 (56.25%) was most frequent in SV, contrasting with NC’s minimal virilization (Normal external genitalia: 83.33%). This phenotypic gradient aligned with elevated ACTH and P levels in SW/SV versus NC girls, though 17OHP and T showed non-significant trends. The higher prevalence of severe virilization (Prader ≥3) in SW versus SV girls may reflect comparatively elevated testosterone in this subgroup, consistent with prior reports (24, 28).

Critically, virilized girls (Prader ≥1; 90.20%) demonstrated accelerated diagnosis timelines and significantly higher ACTH, 17OHP, and progesterone. Surgical intervention occurred earlier and more frequently with increasing severity–92.59% of Prader ≥3 girls underwent surgery at median age 2.10 years versus 57.89% at 3.80 years in Prader 1~2. The overall surgical rate (78.26%) was slightly lower than previously reported (85%) (29). Studies demonstrated that patients undergoing surgery after 2 years of age required significantly fewer additional major surgeries and had higher long-term satisfaction rates than those operated on before 2 years of age (30). No significant association was observed between Prader scores and postnatal steroid hormone levels in this study. This may be attributed to several factors: postnatal steroid concentrations are susceptible to confounding variables such as stress and may not accurately reflect peak androgen exposure during the critical window of urogenital sinus differentiation. Additionally, individual tissue responsiveness to androgens is influenced by factors such as 5α-reductase activity, androgen receptor sensitivity, and coregulator expression (31, 32). Finally, the scope of androgen assessment was limited; key biomarkers including free testosterone, dihydrotestosterone, and 11-oxygenated androgens were not analyzed (33).

Our MLPA assay, employing six *TNXB*-specific probes, identified exon 35 deletions in 30/106 patients (28.30%). Of these, 12 patients (11.3% overall; 40% of *CYP21A2*-deleted cases) harbored contiguous deletions extending from *CYP21A2* to *TNXB* exon 35, generating *TNXA/TNXB* chimeras (exons 1–34 of *TNXB* + exons 35–44 of *TNXA*) diagnostic of CAH-X CH-1. This prevalence exceeds prior reports (7–8.2%) (8, 11), suggesting population-specific variability, and chimeric mutations demonstrate incomplete penetrance for EDS features (8, 11), mild EDS manifestations may occur even without chimeras (14). Patients with missense-driven CH-2/3 exhibit greater joint hypermobility than CH-1 cases (8), likely reflecting distinct pathomechanisms: CH-1 causes an extracellular matrix protein TNX haploinsufficiency, whereas CH-2/3 exert dominant-negative effects (12, 13). A limitation of this study is the absence of systematic EDS assessments and lack of *TNXB* exons 40, 41, and 43 sequencing.

Genotype-phenotype correlations demonstrated high PPV for severe phenotypes (Group 0: 100%; Group A: 87.50%), while moderate PPV was observed for Groups B (76.92%) and C (75.00%). This largely aligns with established literature, although the PPV of Group B varies across studies involving different populations (10, 34–36), suggesting greater phenotypic heterogeneity in moderate forms. Such variability likely stems from modifier genes influencing 21-hydroxylase expression and steroidogenesis (4, 22), as well as divergent genetic backgrounds among different ethnic groups, further supporting the clinical continuum observed among the three phenotypes.

Molecular profiling revealed three variant classes: micro-conversions predominated (70.89%), followed by large deletions/conversions (19.25%) and bona fide point mutations (9.86%), consistent with Asian populations (22, 37). The first three common variants were: I2G (31.46%); large deletions/conversions (19.25%) and p.I173N (13.62%). The first three common variants were consistent with the most common variants in Asian populations (9, 22, 24, 37). It should be noted that the MLPA approach used here, while effective for confirming deletions, may miss complex rearrangements and does not provide breakpoint resolution. Further analysis with advanced sequencing methods is warranted to fully elucidate the structure of these variants in the future.

Notably, p.V282L frequency (5.16%) exceeded previous similar reports yet remained below Argentinian/high-admixture cohorts (23.9–26.2%) (36, 38), indicating ethnogeographic variation. The p.V282L variant dominated our NC subgroup (5/9 cases), including two homozygotes, establishing it maybe a regional hotspot. While p.V282L and p.P31L share comparable residual activity *in vitro*, p.P31L carriers exhibit poorer genotype-phenotype concordance and higher symptom penetrance (4, 29)—consistent with its rarity (1/9 NC patients) in our cohort.

Two male patients (1.89%) in Group D exhibited monoallelic variants: one NC phenotype with I2G and one SW with p.Q319X, despite comprehensive screening revealing no second pathogenic allele. This aligns with reported monoallelic detection rates (2.2–24%) (4, 10, 34, 36, 39), potentially relating to pseudogenes affecting amplification or some unidentified intron region variants affecting transcriptional activity (24, 40). Notably, three male patients (2.83%) harbored paternally inherited *cis* double mutations: two brothers with discordant SW/SV phenotypes carried the first-reported discontinuous mutations (E3del8bp & p.V282L), while one SW patient had adjacent mutations (p.L308Ffs*6 & p.Q319X) previously hypothesized to be minor conversions (4, 24, 39, 41). The phenotypic divergence between siblings sharing identical paternal mutations implies the contribution of factors beyond the primary genetic defect, such as modifier genes, epigenetic influences, or stochastic developmental processes, which alter enzyme activity, hormone levels, or tissue responsiveness. Future studies employing genome-wide approaches and family-based designs are warranted to elucidate the complex interplay between primary mutations, modifier genes, and epigenetics in shaping the phenotypic landscape of 21OHD (38).

Additionally, three patients (2.83%) possessed *de novo* mutations absent in maternal blood, suggesting germline mosaicism (4). This incidence exceeds the 1% literature baseline (4), underscoring *de novo* events as non-trivial contributors. Finally, we validated a novel splice variant (c.203-1G>A) in an SW female. Splice-AI predicted exon skipping (probability=0.99), confirmed *in vitro* to cause exon 2 exclusion (NM_000500.9: c.203_292del) and protein truncation, explaining the severe phenotype when co-occurring with a deletion allele.

## 5 Conclusions

In conclusion, this study comprehensively analyzed clinical phenotypes, steroid hormone profiles, and molecular genetics in 115 Chinese patients with 21OHD. We identified and characterized a novel pathogenic splicing mutation (c.203-1G>A), demonstrating it induces exon 2 skipping during mRNA processing. We report the first documented case of paternally inherited, discontinuous *cis* double mutations (E3del8bp and p.V282L) in siblings exhibiting an intriguing discordant SW/SV phenotype. Significantly elevated levels of ACTH, AD, and P were demonstrated in the SW and SV groups compared to the NC group. Girls with vulvar masculinization exhibited higher ACTH, 17OHP, and P levels than non-virilized girls, and severe virilization was more prevalent in the SW than SV group. NBS substantially reduced time-to-diagnosis, particularly in male SW infants, underscoring the value of implementing nationwide NBS in China. We established genotype-phenotype correlations and defined the variant frequency, confirming I2G, large deletions/conversions, and p.I173N as predominant *CYP21A2* mutations in this Chinese cohort. Notably higher frequencies of p.V282L and CAH-X CH-1 compared to previous reports suggest enhanced clinical recognition, particularly of NC form.

## Data Availability

The datasets presented in this study can be found in online repositories. The names of the repository/repositories and accession number(s) can be found in the article/**Supplementary Material**.
