# Supplementary material for: Comprehensive characterization of 21-hydroxylase deficiency in a Chinese pediatric cohort: phenotype, steroid profiles and genetics
Source: Front Endocrinol (Lausanne). 2025 Oct 16;16:1665306. doi: 10.3389/fendo.2025.1665306 (PMC12571618; doi:10.3389/fendo.2025.1665306)
Supplement: Supplementary file 1 [file DataSheet1.zip › Supplementary Table 2.DOCX]

**Table S2 Phenotype- and Sex-Stratified Profiles of the 100 Chinese children with 21OHD** **at initial diagnosis**

|  |  | SW |  |  |  | SV |  |  |  | NC |  |
| --- | --- | --- | --- | --- | --- | --- | --- | --- | --- | --- | --- |
|  | Male  (n=39) | Female  (n=27) | Total  (n=66) |  | Male  (n=10) | Female  (n=12) | Total  (n=22) |  | Male  (n=6) | Female  (n=6) | Total  (n=12) |
| Sym age (yr) | 0.03  (0.01;0.06) ^ac*^ | 0.00  (0.00;0.00) ^a^ | 0.01  (0.00;0.04) ^a^^c^ |  | 3.76  (1.02;6.71) ^*^ | 0.00  (0.00;0.85) ^b^ | 0.66  (0.00;4.42) ^b^ |  | 3.96  (2.92;6.10) ^*^ | 11.85  (7.65;14.64) | 7.43  (3.72;12.68) |
| Sym-diag  interval (yr) | 0.04  (0.01;0.10) ^a^ | 0.06  (0.04;0.20) ^ac^ | 0.05  (0.02;0.11) ^ac^ |  | 0.55  (0.02;4.43) | 2.37  (0.34;4.31) | 1.69  (0.18;4.28) |  | 3.81  (1.72;4.69) | 1.23  (0.37;3.29) | 2.72  (0.50;4.22) |
| Diag age (yr) | 0.08  (0.05;0.15) ^ac^ | 0.06  (0.04;0.20) ^ac^ | 0.08  (0.05;0.16) ^ac^ |  | 6.98  (2.35;8.37) | 2.90  (0.34;5.70) ^b^ | 4.72  (0.39;8.35) ^b^ |  | 7.24  (6.73;9.59) ^*^ | 13.49  (8.16;16.92) | 8.91  (6.98;13.56) |
| Na+  (mmol/L) | 119.40  (108.00;  129.80) ^ac^ | 122.60  (113.00;  131.00) ^ac^ | 121.35  (111.10;  131.00) ^ac^ |  | 138.00  (133.95;  139.05) | 137.30  (131.93;  139.75) | 137.80  (133.58;  139.20) ^b^ |  | 138.00  (102.45;  140.03) | 140.15  (138.58;  142.80) | 139.20  (137.25;  141.80） |
| K+  (mmol/L) | 6.80  (5.93;7.86) ^ac^ | 6.30  (5.73;7.60) ^ac^ | 6.58  (5.80:7.82) ^ac^ |  | 4.43  (3.97;4.94) | 4.65  (4.42;4.94) ^b^ | 4.60  (4.18;4.91) ^b^ |  | 4.15  (3.06;4.30) | 4.12  (3.96;4.26) | 4.15  (4.00;4.28) |
| ACTH  (pg/ml) | 259.90  (113.00;  892.30) | 362.80  (135.00;  1250.10) ^a^ | 281.20  (113.90;  906.25) ^a^ |  | 121.50  (80.23;  422.75) | 180.70  (81.35;  296.45) | 137.70  (80.23;  311.50) ^b^ |  | 71.39  (22.75;  133.63) | 79.70  (20.30;  124.50) | 79.70  (22.25;  119.75) |
| 17OHP  (ng/ml) | 129.80  (63.07;  232.10) | 178.20  (50.80;  278.00) | 135.76  (60.00;  263.03) ^a^ |  | 87.10  (71.53;  162.40) | 37.46  (26.35;  174.15) | 78.90  (32.37;  137.13) ^b^ |  | 19.50  (11.51;  66.43) | 70.73  (2.99;  146.87) | 37.00  (5.91:  89.84） |
| T  (pg/ml) | 3870.00  (1780.00;  7381.90) | 4330.00  (2820.00;  8900.00) | 3978.75  (2245.75;  7720.00) ^ac^ |  | 1670.00  (745.58;  2609.00) | 2687.00  (1207.28;  4814.05) | 2060.05  (958.28;  3635.05) |  | 926.09  (227.50;  3180.00) | 1838.60  (265.28;  5392.50) | 1499.04  (249.53;  3366.80) |
| P  (ng/ml) | 13.26  (5.82;20.50)^a^ | 14.24  (7.36;25.02) ^a^ | 13.57  (7.33;22.45)^a^ |  | 6.00  (1.33;21.25) | 12.46  (7.20;20.10) ^b^ | 10.54  (4.71;17.57) ^b^ |  | 1.64  (0.27;5.31) | 2.13  (0.91;6.87) | 2.13  (0.37:4.28) |

Different superscript letters (a, b, c) indicate statistically significant differences (*P*<0.05) between phenotypes: a, SW vs NC; b, SV vs NC; c, SW vs SV. Asterisk (*) indicates significant difference (*P*<0.05) between males and females within the same phenotype group.

Sym age: age at symptom onset. Sym-diag interval: symptom-to-diagnosis interval. Diag age: age at diagnosis. yr:years-old.
