# Supplementary material for: Comprehensive characterization of 21-hydroxylase deficiency in a Chinese pediatric cohort: phenotype, steroid profiles and genetics
Source: Front Endocrinol (Lausanne). 2025 Oct 16;16:1665306. doi: 10.3389/fendo.2025.1665306 (PMC12571618; doi:10.3389/fendo.2025.1665306)
Supplement: Supplementary file 1 [file DataSheet1.zip › Supplementary Table 1.DOCX]

**Table S1 Clinical characteristics of the 9 patients without genetic testing**

|  | Sex | Chief complaint | Sym age | Diag age | ACTH  (pg/ml) | cortisol (ng/ml) | Na^+^  (mmol/L) | K^+^  (mmol/L) | 17OHP  (ng/ml) | AD  (pg/ml) | T  (pg/ml) | P  (ng/ml) | 21DOF  (pg/ml) | Phen-otype |
| --- | --- | --- | --- | --- | --- | --- | --- | --- | --- | --- | --- | --- | --- | --- |
| P1 | F | Hyperpigmentation,  atypical genitalia,  drowsiness | 0d | 15d | 257.80 | 36.30 | 119.00 | 7.90 | 109.30 | NA | 9210.00 | 25.30 | NA | SW |
| P2 | M | Hyperpigmentation,  emesis, drowsiness | 2d | 15d | 192.80 | 32.60 | 121.00 | 7.07 | 75.10 | 7780.00 | 11200.00 | 8.98 | NA | SW |
| P3 | F | Hyperpigmentation,  atypical genitalia,  emesis, drowsiness | 0d | 3d | 532.00 | 14.80 | 122.60 | 8.41 | 135.10 | NA | 16600.10 | 40.10 | NA | SW |
| F4 | F | Hyperpigmentation, atypical genitalia,  emesis, seizure | 0d | 2d | >1250.00 | NA | 101.30 | 7.20 | 214.50 | 9870.00 | 7230.00 | 3.20 | NA | SW |
| P5 | M | Accelerated growth,  penile enlargement and thickening,  acne,  premature pubarche | 2.5yr | 2.5yr | 132.00 | 34.80 | 136.00 | 4.32 | 88.50 | 4690.00 | 3870.00 | 4.49 | 15277.00 | SV |
| P6 | F | atypical genitalia,  premature pubarche | 0.3yr | 5.4yr | 248.70 | 36.10 | 138.00 | 4.46 | 109.00 | 9820.00 | 2640.00 | 6.63 | NA | SV |
| P7 | F | Irregular menstruation,  hirsutism | 13yr | 17yr | 46.00 | Basal:25.60  Peak:102.50 | 140.60 | 3.85 | Basal: 2.82  Peak: 21.35 | NA | 2817.20 | 4.37 | NA | NC |
| P8 | F | Irregular menstruation,  hirsutism | 13yr | 15yr | 58.30 | Basal:47.20  Peak:98.41 | 142.20 | 4.33 | Basal: 3.12  Peak: 19.62 | 857.60 | 910.50 | 3.78 | 1051.10 | NC |
| P9 | M | Accelerated growth,  advanced bone age | 5yr | 8.7yr | 107.00 | Basal:32.80  Peak: NA | 138.30 | 4.10 | Basal: 5.78  Peak: 28.37 | 1663.80 | 783.20 | 1.12 | 2307.30 | NC |

F: female. M: male. Sym age: age at symptom onset. Diag age: age at diagnosis. d: days old. yr: years-old. NA: not available. The ACTH stimulation test was performed to measure serum concentrations of cortisol and 17OHP at baseline and 60 minutes following intravenous administration of ACTH.
