# Supplementary material for: Comprehensive characterization of 21-hydroxylase deficiency in a Chinese pediatric cohort: phenotype, steroid profiles and genetics
Source: Front Endocrinol (Lausanne). 2025 Oct 16;16:1665306. doi: 10.3389/fendo.2025.1665306 (PMC12571618; doi:10.3389/fendo.2025.1665306)
Supplement: Supplementary file 1 [file DataSheet1.zip › Supplementary Figure 1.DOCX]

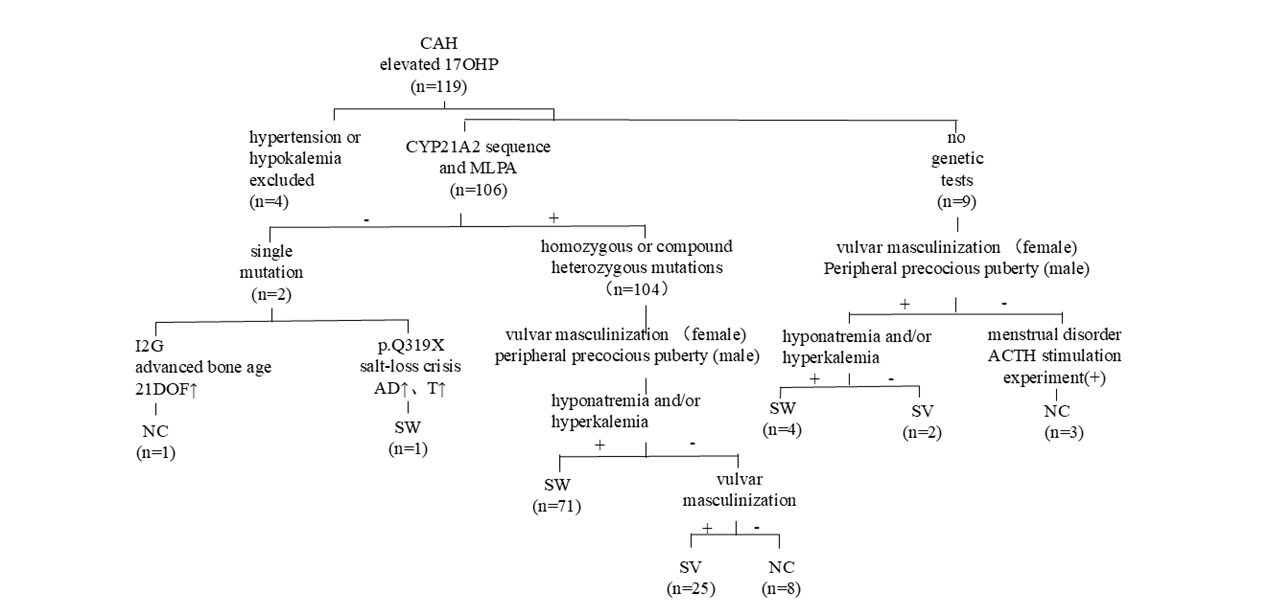


**Figure.S1. Inclusion criteria**

A cohort of 119 cases with clinical and biochemical features of CAH—including hyperpigmentation, hypocortisolism, elevated ACTH, vomiting, atypical genitalia—along with elevated 17-OHP was collected. Four cases presenting hypertension or hypokalemia were excluded; all were confirmed by genetic testing to carry compound heterozygous mutations in *CYP11B1*.Among the remaining cases, 106 underwent *CYP21A2* sequencing and MLPA analysis. Two carried only a single heterozygous mutation (one SW and one NC), while 104 had homozygous or compound heterozygous mutations, which were classified as SW (n = 71), simple virilizing (SV) (n = 25), or NC (n = 8) based on clinical and electrolyte profiles. The remaining nine cases, without genetic testing, were phenotypically categorized as SW (n = 4), SV (n = 2), or NC (n = 3).
