# Supplementary material for: Comprehensive characterization of 21-hydroxylase deficiency in a Chinese pediatric cohort: phenotype, steroid profiles and genetics
Source: Front Endocrinol (Lausanne). 2025 Oct 16;16:1665306. doi: 10.3389/fendo.2025.1665306 (PMC12571618; doi:10.3389/fendo.2025.1665306)
Supplement: Supplementary file 1 [file DataSheet1.zip › Supplementary Figure 7.DOCX]

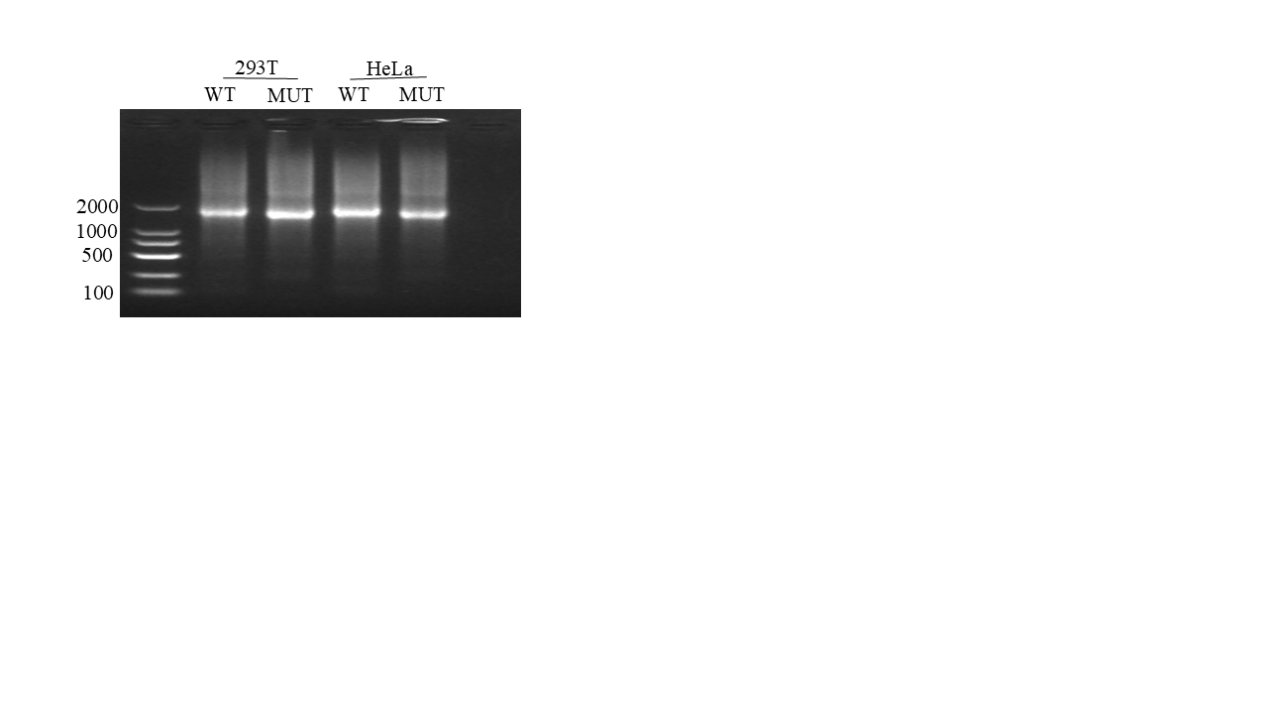


Figure S7. Gel electrophoretogram of nested PCR

The graph shows the results of nested PCR after transfection of wild-type and mutant plasmids in 293T cells and HeLa cells from left to right. The bands of the transfected wild-type plasmid were slightly higher than those of the mutant plasmid in both cells.
