# Supplementary material for: Comprehensive characterization of 21-hydroxylase deficiency in a Chinese pediatric cohort: phenotype, steroid profiles and genetics
Source: Front Endocrinol (Lausanne). 2025 Oct 16;16:1665306. doi: 10.3389/fendo.2025.1665306 (PMC12571618; doi:10.3389/fendo.2025.1665306)
Supplement: Supplementary file 1 [file DataSheet1.zip › Supplementary Figure 6.DOCX]

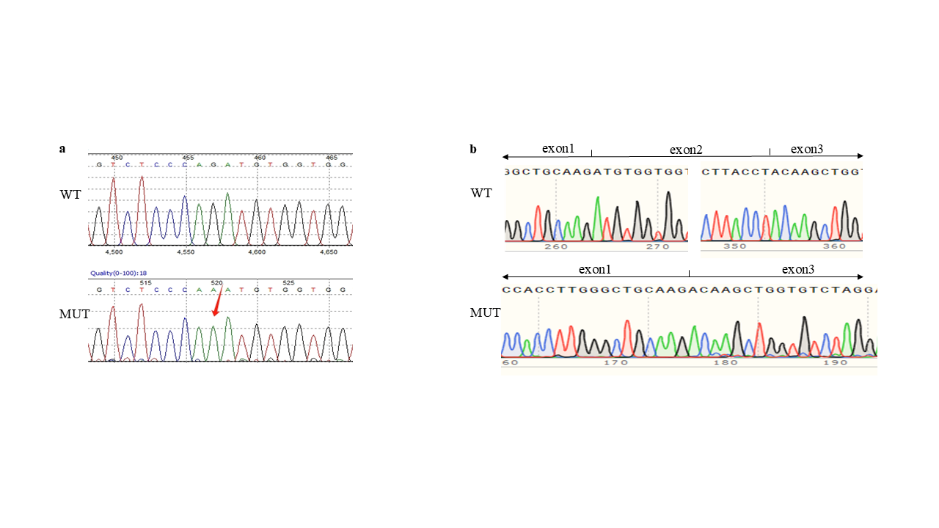


Figure S6. Sanger sequencing of novel variants in the CYP21A2 gene

1. Sanger sequencing of splice mutation c.203-1G>A, red arrows indicate mutation location and type
2. CYP21A2 transcripts Sanger sequencing: the entire 90 bp sequence of exon2 is lost in the mutant mRNA, and the shortened mRNA is indicated as NM_000500.9:c.203_292del.WT: wild-type. MUT: mutant-type.
