# Supplementary material for: Comprehensive characterization of 21-hydroxylase deficiency in a Chinese pediatric cohort: phenotype, steroid profiles and genetics
Source: Front Endocrinol (Lausanne). 2025 Oct 16;16:1665306. doi: 10.3389/fendo.2025.1665306 (PMC12571618; doi:10.3389/fendo.2025.1665306)
Supplement: Supplementary file 1 [file DataSheet1.zip › Supplementary Figure 5.DOCX]

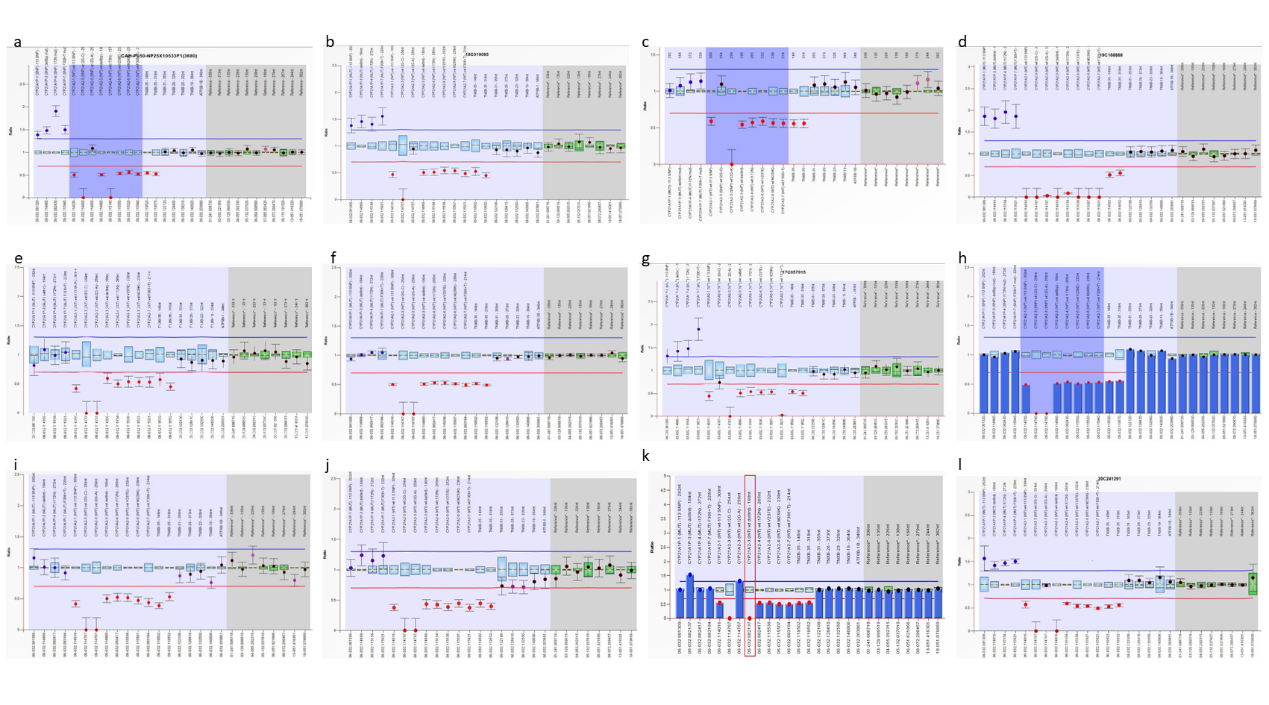


Figure S5. MLPA sequencing of 12 patients with CAH-X syndrome

Figures a-l sequentially represent the MLPA sequencing results of 12 patients with CAH-X syndrome.
