# Supplementary material for: Comprehensive characterization of 21-hydroxylase deficiency in a Chinese pediatric cohort: phenotype, steroid profiles and genetics
Source: Front Endocrinol (Lausanne). 2025 Oct 16;16:1665306. doi: 10.3389/fendo.2025.1665306 (PMC12571618; doi:10.3389/fendo.2025.1665306)
Supplement: Supplementary file 1 [file DataSheet1.zip › Supplementary Figure 4.DOCX]

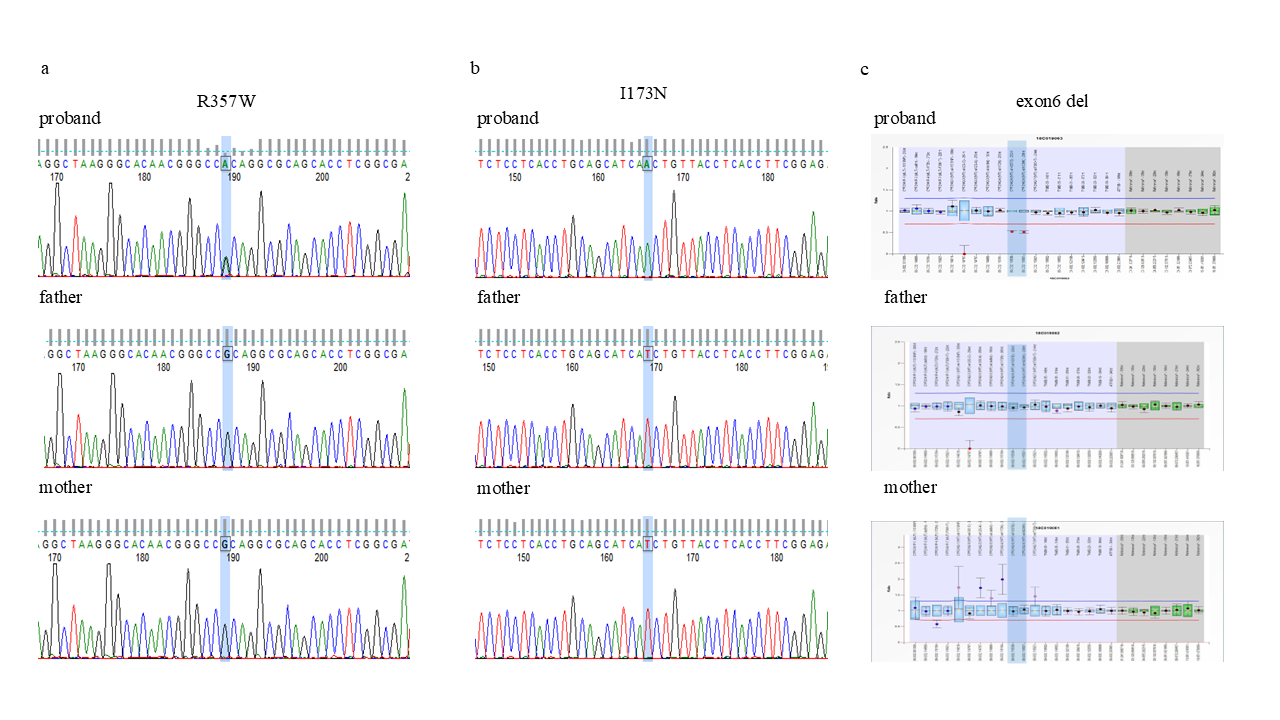


Figure S4. Lineage validation of de novo mutations

The blue boxes indicate the location and type of mutations, and figures a-c indicate the lineage validation of the probands carrying the mutations in R357W, I173N, and exon6 deletion respectively, whose parents did not carry the corresponding mutation.
