# Supplementary material for: Comprehensive characterization of 21-hydroxylase deficiency in a Chinese pediatric cohort: phenotype, steroid profiles and genetics
Source: Front Endocrinol (Lausanne). 2025 Oct 16;16:1665306. doi: 10.3389/fendo.2025.1665306 (PMC12571618; doi:10.3389/fendo.2025.1665306)
Supplement: Supplementary file 1 [file DataSheet1.zip › Supplementary Figure 3.DOCX]

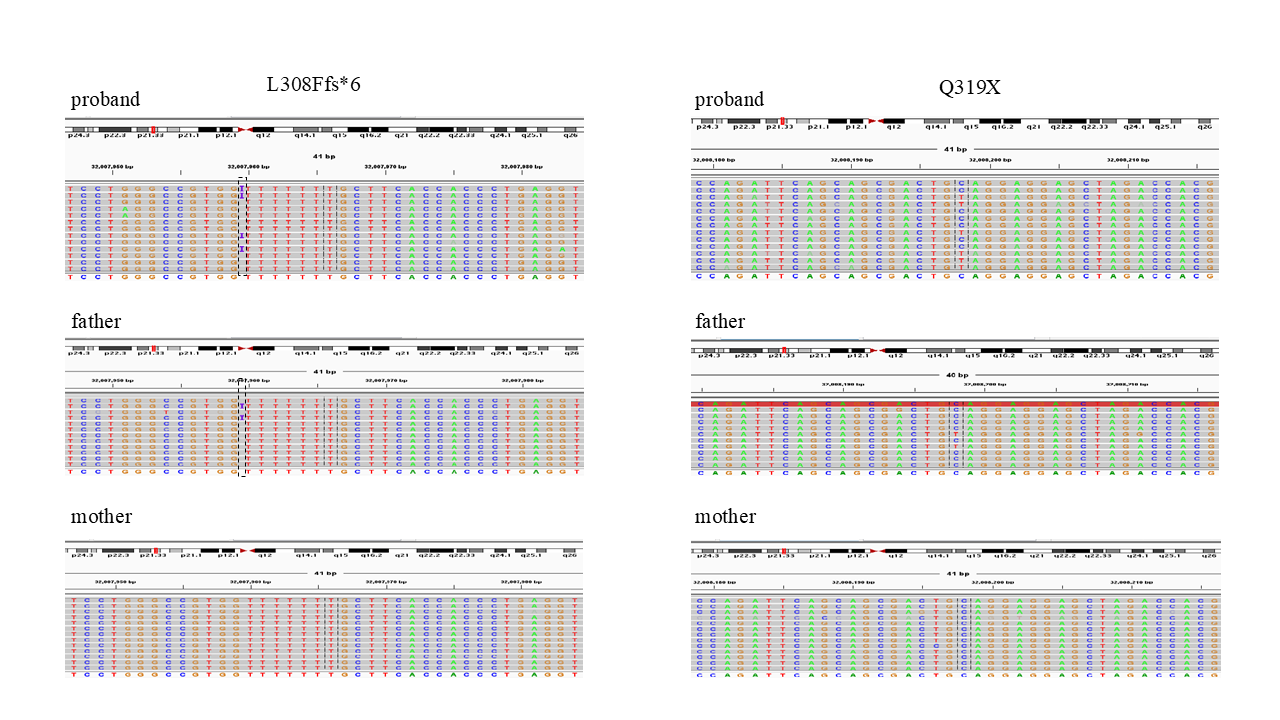


Figure S3. *Cis* double mutations of L308Ffs*6 & Q319X

The dashed boxes show the location and type of mutations. Both the proband and his father carry the L308Ffs*6 mutation (Figure S3a) and the Q319X mutation (Figure S3b), while his mother does not carry either of these mutations.
