# Supplementary material for: Comprehensive characterization of 21-hydroxylase deficiency in a Chinese pediatric cohort: phenotype, steroid profiles and genetics
Source: Front Endocrinol (Lausanne). 2025 Oct 16;16:1665306. doi: 10.3389/fendo.2025.1665306 (PMC12571618; doi:10.3389/fendo.2025.1665306)
Supplement: Supplementary file 1 [file DataSheet1.zip › Supplementary Figure 2.DOCX]

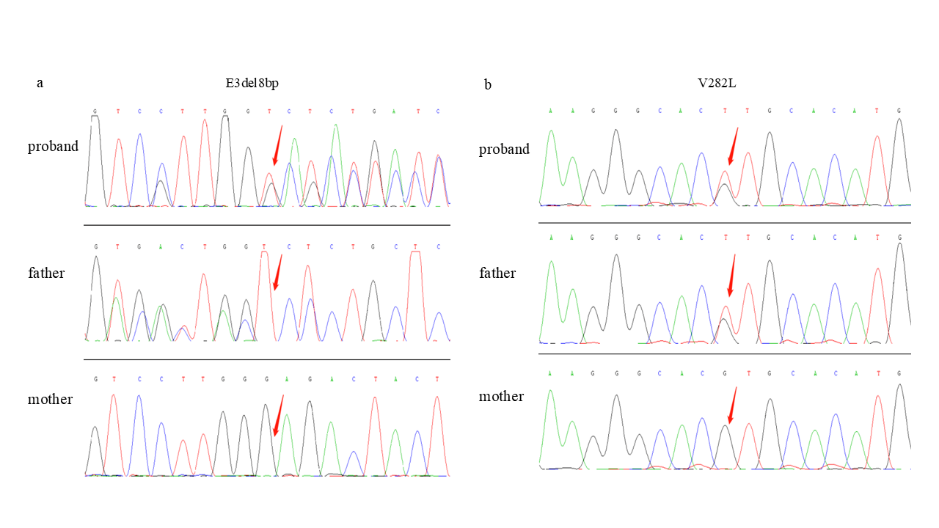


Figure S2. Double mutations of E3del8bp and V282L

The red arrows show the location and type of mutations. Both the proband and his father carry the E3del8bp mutation (Figure S2a) and the V282L mutation (Figure S2b), while his mother does not carry either of these mutations.
